# Somatic alterations compromised molecular diagnosis of DOCK8 hyper-IgE syndrome caused by a novel intronic splice site mutation

**DOI:** 10.1038/s41598-018-34953-z

**Published:** 2018-11-13

**Authors:** Beate Hagl, Benedikt D. Spielberger, Silvia Thoene, Sophie Bonnal, Christian Mertes, Christof Winter, Isaac J. Nijman, Shira Verduin, Andreas C. Eberherr, Anne Puel, Detlev Schindler, Jürgen Ruland, Thomas Meitinger, Julien Gagneur, Jordan S. Orange, Marielle E. van Gijn, Ellen D. Renner

**Affiliations:** 1 Chair and Institute of Environmental Medicine, UNIKA-T, Technical University of Munich and Helmholtz Zentrum Munich, Munich/Augsburg, Munich, Germany; 2 University Children’s Hospital, Dr. von Haunersches Kinderspital, Ludwig Maximilian University, Munich, Germany; 3 Institute of Clinical Chemistry and Pathobiochemistry, Klinikum rechts der Isar, Technical University of Munich, Munich, Germany; 4 German Cancer Consortium (DKTK), partner site Munich, Munich, Germany; 5 German Cancer Research Center (DKFZ), Heidelberg, Germany; 6 Centre for Genomic Regulation (CRG), The Barcelona Institute of Science and Technology, Dr. Aiguader 88, Barcelona, 08002 Spain; 7 Universitat Pompeu Fabra (UPF), Barcelona, Spain; 8 Department of Informatics, Technical University of Munich, Garching, Germany; 9 Department of Genetics, University Medical Center Utrecht, Utrecht, The Netherlands; 10 Laboratory of Human Genetics of Infectious Diseases, Necker Branch, Necker Medical School, Paris, France; 11 Paris Descartes University, Sorbonne Paris Cité, Institut Imagine, Paris, France; 12 St Giles Laboratory of Human Genetics of Infectious Diseases, Rockefeller Branch, Rockefeller University, New York, NY USA; 13 Department of Human Genetics, University of Würzburg, Würzburg, Germany; 14 German Center for Infection Research (DZIF), partner site Munich, Munich, Germany; 15 Institute of Human Genetics, Technical University of Munich and Helmholtz Zentrum Munich, Neuherberg, Germany; 16 Quantitative Biosciences Munich, Gene Center, Department of Biochemistry, Ludwig Maximilian University, Munich, Germany; 17 Center for Human Immunobiology of Texas Children’s Hospital/Department of Pediatrics, Baylor College of Medicine, Houston, TX USA; 18 Department of Pediatrics, Division of Immunology, Allergy, and Rheumatology, Baylor College of Medicine, and Texas Children’s Hospital, Houston, TX USA; 19 Department of Pediatrics, Baylor College of Medicine, and Texas Children’s Hospital, Houston, TX USA; 20 Hochgebirgsklinik and Christine-Kühne-Center for Allergy Research and Education (CK-Care), Davos, Switzerland

## Abstract

In hyper-IgE syndromes (HIES), a group of primary immunodeficiencies clinically overlapping with atopic dermatitis, early diagnosis is crucial to initiate appropriate therapy and prevent irreversible complications. Identification of underlying gene defects such as in *DOCK8* and *STAT3* and corresponding molecular testing has improved diagnosis. Yet, in a child and her newborn sibling with HIES phenotype molecular diagnosis was misleading. Extensive analyses driven by the clinical phenotype identified an intronic homozygous *DOCK8* variant c.4626 + 76 A > G creating a novel splice site as disease-causing. While the affected newborn carrying the homozygous variant had no expression of DOCK8 protein, in the index patient molecular diagnosis was compromised due to expression of altered and wildtype *DOCK8* transcripts and DOCK8 protein as well as defective STAT3 signaling. Sanger sequencing of lymphocyte subsets revealed that somatic alterations and reversions revoked the predominance of the novel over the canonical splice site in the index patient explaining DOCK8 protein expression, whereas defective STAT3 responses in the index patient were explained by a T cell phenotype skewed towards central and effector memory T cells. Hence, somatic alterations and skewed immune cell phenotypes due to selective pressure may compromise molecular diagnosis and need to be considered with unexpected clinical and molecular findings.

## Introduction

Patients with primary immunodeficiencies (PIDs), such as hyper-IgE syndromes (HIES), have benefited tremendously from clinical classifications and the discovery of underlying gene defects and corresponding molecular testing. HIES are rare immunodeficiencies characterized by eczema, elevated serum IgE levels, eosinophilia and recurrent infections; and depending on the underlying genetic defect, additionally persistent primary teeth, allergic findings, lymphopenia or low Th17 cell counts^1–7^. All HIES entities overlap significantly with more common diseases, particularly severe forms of atopic dermatitis. Hence, prior to the possibility of molecular testing and due to low awareness of HIES, diagnosis was often delayed until severe complications, particularly irreversible lung changes, have impacted patients’ quality of life.

The identification of genes causing HIES enabled diagnostic blood testing of low Th17 cell counts and reduced STAT3 phosphorylation in STAT3-HIES^8,9^, or lack of DOCK8 protein expression in DOCK8-HIES^10^. The improved understanding of the immunopathology resulted in treatment optimization^1–4^, such as the benefit of immunoglobulin substitution therapy in addition to rigorous antibiotic treatments due to the discovered impaired adaptive immunity^5,11^ and the consensus of early hematopoietic stem cell transplantation (HSCT) as treatment of choice in HIES caused by DOCK8 deficiency (DOCK8-HIES)^11–13^.

With the knowledge that early diagnosis often determines the disease outcome, targeted next-generation sequencing (NGS) has become a cost-efficient tool in PID diagnostics^14,15^. Due to the rapidly increasing number of newly defined monogenic PIDs molecular PID diagnostics already needs to cover over 350 genes and targeted NGS approaches are limited to a pre-defined set of disease-causing genes^15,16^. Therefore, whole exome sequencing (WES) and whole genome sequencing (WGS) analyses are starting to replace targeted approaches^15^.

The experience of the following family shows how somatic alterations complicated molecular diagnosis and how the close interplay of clinical, immunological, molecular, and bioinformatic diagnostic approaches identified a genetically determined disease.

## Results

### Clinical and immunological presentation

**Figure 1:**
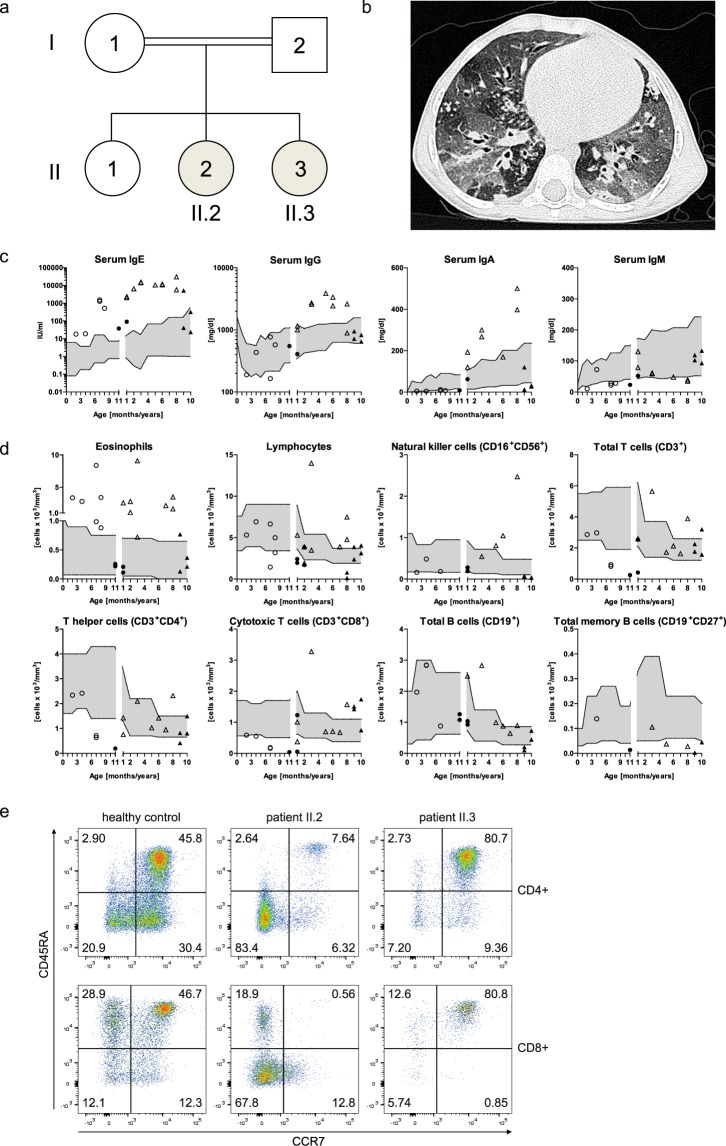
Pedigree, clinical and immunologic presentation of the two affected siblings. (**a**) Pedigree of the investigated consanguineous family of the two affected siblings: II.2: index patient; II.3: second affected child. (**b**) Chest CT scan of patient II.2 at 3.5 years of age with areas of ground glass opacities, air trapping, multiple irregular nodular opacities (tree-in-bud sign), and bilateral bronchiectasis representing lung parenchyma destruction. (**c**) Serum immunoglobulin levels, (**d**) absolute numbers of eosinophil, lymphocyte and lymphocyte subsets of patient II.2 (triangles) and patient II.3 (circles) before (open symbols) and after HSCT (black filled symbols) compared to age-related normal range (gray filled area). (**e**) Representative flow cytometric plots showing CD4^+^ and CD8^+^ T cell subsets assessed by CCR7 and CD45RA expression in patient II.2 and patient II.3 compared to a healthy control. Percentages of naïve T cells (CCR7^+^CD45RA^+^), central memory T cells (CCR7^+^CD45RA^−^), effector memory T cells (CCR7^−^CD45RA^−^) and T_EMRA_ cells (CCR7^−^CD45RA^+^) of CD4^+^ or CD8^+^ T cells are indicated by numbers in the respective quadrant.

The index patient (patient II.2) is the second child of healthy first-degree cousins (Fig. 1a); term born with 2860 g (7^th^ percentile) and 34 cm head circumference (25^th^ percentile) after uneventful pregnancy. Recurrent upper and lower respiratory infections started at 2 months, including a fulminant pneumonia, requiring intubation and ventilation resulting in bronchiolitis obliterans at 6 months of age. Recurrent infections led to additional chronic lung changes with bronchiectasis formation and *Pseudomonas aeruginosa* positive lung specimen following frequent exacerbations (Fig. 1b). She developed eczema at 4 months with generalized eczema herpeticatum at 12 months of age. Several flares of eczema herpeticatum followed, including a herpes simplex blepharitis at around 8 years of age. There were repeated *Molluscum contagiosum* and mucocutaneous Candida infections as well as recurrent onychomycosis. She developed multiple specific IgE positive food allergies to milk, eggs, soy, and peanuts; at 12 months of age she suffered an anaphylactic reaction to lentils. Her growth started to slow to the 3^rd^ percentile at 6 months of age and dropped further with significant growth retardation, malnutrition, and iron deficient anemia.

At 3.5 years of age she was referred for PID evaluation. HIES was suspected due to elevated serum IgE (max. value 30434 IU/ml), eosinophilia (max. value 9100 cells/µl), chronic eczema, and recurrent bacterial, fungal, and viral infections, particularly of skin and lungs. Except for hyperextensible joints, she had no other skeletal findings associated with STAT3-HIES. The overall clinical presentation was in-between STAT3- and DOCK8-HIES with 48 points in the NIH-HIES score^17^, which sums up findings of STAT3-HIES and is considered predictive for HIES above 40 points.

Immunological work-up revealed high IgE, IgG, and IgA levels, and low to normal IgM levels (Fig. 1c). Mainly normal or high total lymphocyte and NK cell counts with high eosinophil counts were measured (Fig. 1d). Memory B cell counts affecting total, switched and unswitched memory B cells were low. Absolute numbers of total T cells, T helper cells, and cytotoxic T cells were within normal range, except for individual low and high values. Considering the patient’s young age, her T cell subsets showed a shift towards a memory phenotype with low percentages of naïve T cells (CCR7^+^CD45RA^+^) and high percentages of effector memory T cells (CCR7^-^CD45RA^-^) in CD4^+^ and CD8^+^ T cells and CD8^+^ T_EMRA_ cells (CCR7^−^CD45RA^+^) (Fig. 1e). Th17 cell counts were normal on two time-points with 0.32% and 0.23% of CD4^+^ cells (normal value: >0.20% of CD4^+^ cells). Lymphocyte stimulation tests assessing proliferation by [^3^H]thymidine uptake had partially reduced response to mitogens such as Phytohaemagglutinin, OKT3, Concanavalin A and Pokeweed mitogen and markedly reduced reactions to an antigen mixture of Tetanus and Diphteria toxoid antigen (data not shown).

Since recurrent infections and severe failure to thrive were not controlled by intensive antibacterial, -viral and -fungal treatment, the patient was referred again at 8 years of age. When the family ultimately agreed to HSCT, she received donor cells of her mother at 8 years of age. After an uneventful HSCT, her general health condition progressed over the next months with significant improvement of eczema and skin infections and restart of weight- and height-gain. Lymphocyte stimulation test to mitogens as well as B and T cell compartments, including naïve T cells, normalized within the first year after HSCT except for a shift towards cytotoxic T cells. Initially recurrent lung infections remained unchanged, most likely due to the bronchiolitis obliterans resulting from the severe infection at 6 months of age. Three years after HSCT, the frequency of respiratory infections has improved while continuous antibiotic and inhalation treatment are still required. Food allergies to milk, egg, and nuts were still present.

Shortly after HSCT of patient II.2, a third child (patient II.3) was born (3790 g of weight; 77^th^ percentile; 36.5 cm head circumference; 89^th^ percentile). Within the first months of age, she developed eczema, hard to control skin infections, and chronic otitis externa. Except for slightly elevated serum IgE (initially 18.2 IU/ml; max. value 1532 IU/ml at 7 months) and eosinophilia, she presented with lymphocyte counts and proliferation unremarkable for age (data not shown). Also NK, T and B cell counts, including subpopulations and IgG, IgA, and IgM serum were unremarkable for age (Fig. 1c–e). At 8 months of age, she received HSCT from a healthy unrelated donor. So far, she has developed completely normal without eczema, skin or unusual respiratory infections more than two years after HSCT.

### STAT3 signaling analyses

**Figure 2:**
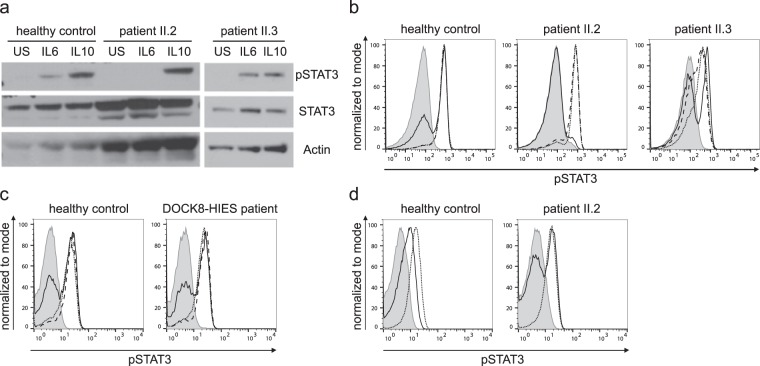
STAT3 phosphorylation analysis after stimulation. (**a**) Western blot analysis of whole cell lysates of PBMCs, unstimulated or 20 min. stimulated with 200 ng/ml IL6 or IL10. Expression of STAT3 phosphorylated at Y705 (pSTAT3) and total STAT3 (STAT3) of the two affected siblings and a healthy control was assessed; Actin as loading control. (**b**) Representative flow cytometric analysis showing diminished Y705-STAT3 phosphorylation after 20 min. stimulation with 200 ng/ml IL6 (solid line) versus unremarkable results after stimulation with 20 ng/ml IL10 (dotted line) and 10 ng/ml IL21 (dashed line) in lymphocytes of patient II.2 compared to unremarkable results in patient II.3 and a healthy control; filled gray area: unstimulated lymphocytes. (**c**) Flow cytometric analysis showing Y705-STAT3 phosphorylation after 20 min. stimulation with 20 ng/ml IL6 (solid line) or IL10 (dotted line) and 10 ng/ml IL21 (dashed line) comparable to healthy control in lymphocytes of one (representative of four) DOCK8-HIES patient. (**d**) Restored STAT3 phosphorylation after IL6 stimulation (solid line) in patient II.2 15 months after HSCT compared to unstimulated (filled gray area) and IL10-stimulated (dotted line) lymphocytes.

Patient II.2 showed diminished Y705-STAT3 phosphorylation in PBMCs after IL6 stimulation, yet not after IL10 or IL21 stimulation by western blot and flow cytometric analysis (Fig. 2a,b). There was no altered STAT3 phosphorylation in lymphocytes of patient II.3 and four molecularly-defined DOCK8-HIES patients compared to healthy controls (Fig. 2a–c). Slightly decreased STAT3 phosphorylation in lymphocytes of patient II.3 compared to healthy controls was seen in some flow cytometric experiments (Fig. 2b) but overall similar to the range observed in healthy controls and not as clearly reduced as seen in patient II.3. The STAT3 signaling defect in patient II.2 was restricted to immune cells, since STAT3 phosphorylation after IL6 stimulation in patient’s fibroblasts was comparable to control fibroblasts. We ruled out IL6-, IL6-receptor (IL6R) and gp130-autoantibodies as cause of the STAT3 signaling defect by multiplex analyses with serum antibody concentrations below or comparable to levels found in control sera. Furthermore, pre-incubation of PBMCs of a healthy control with serum of patient II.2 had no effect on IL6- and IL10-induced STAT3 phosphorylation (Supplementary Fig. S1). Similarly to other immunological alterations, STAT3 phosphorylation normalized in lymphocytes of patient II.2 within one year after HSCT (Fig. 2d).

### Genetic analyses

In search of the genetic cause of the underlying PID of patient II.2, we performed a previously described PID targeted NGS approach^14^, including *STAT3* and *DOCK8*. A homozygous single nucleotide variant in the *ATM* gene (c.1010 G > A, p.R337H) was detected and ruled-out to be disease-causing by normal radiation sensitivity testing of fibroblasts of patient II.2 and later a normal *ATM* sequence of patient II.3. Next, we completed WES analysis in patient II.2 and identified a homozygous alteration in *ARHGAP32* (c.6038 G > A, p.R2013H). This alteration was considered disease-causing because ARHGAP32 is a GTPase-activating protein (GAP)^18^ and another GAP, called MgcRacGAP, has been associated with IL6-induced STAT3 phosphorylation in human and murine *in vitro* models^19,20^. However, STAT3 phosphorylation was not restored by overexpressing wildtype ARHGAP32 in patient cells and IL6-induced STAT3 phosphorylation in an ARHGAP32 knock-out HAP1 cell line was intact (Supplementary Fig. S2).

WGS of patient II.2 and the healthy parents and a recessive model approach analysis did not lead to any additional candidates. *De novo* variants analysis of the WGS data revealed four putative candidate variants, which were excluded due to their intergenic location or their location of more than 9 kb up- or downstream of adjacent exons.

**Figure 3:**
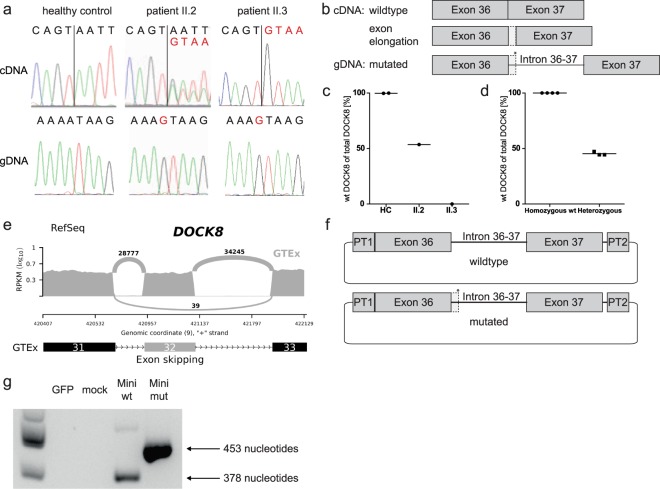
Genetic analysis of *DOCK8*. (**a**) T cell blast cDNA chromatograms show wildtype sequence in a healthy control, double peaks in patient II.2 and altered sequence in patient II.3. Both patients’ gDNA is homozygous for alteration c.4626 + 76 A > G; vertical black lines: 3′ junction of exon 36; black letters: wildtype; red letters: altered sequence. (**b**) Schematic model of affected region in *DOCK8* gDNA and transcripts showing exon extension (dotted line) due to the novel splice site (*) introduced at c.4626 + 76 A > G; filled boxes: exons; horizontal line: intronic region. (**c**) Quantification of wildtype and altered transcripts in T cell blasts by ddPCR indicating percentages of wildtype (wt) of total *DOCK8* transcripts in patient II.2, patient II.3 and healthy controls (HC). (**d**) ddPCR analysis of healthy controls (homozygous wt) and healthy carriers of a c.3120 + 1 G > T *DOCK8* alteration resulting in exon 25 skipping (heterozygous). (**e**) Sashimi plot of RNA sequencing data based on GTEx samples^21,22^ showing exon 32 skipping as a rare event; read counts accumulated over all samples. (**f**) Schematic model of wildtype and mutated minigene vectors. Sequence tags (PT1/PT2) flanked the minigene sequence to differentiate minigene transcripts from endogenous *DOCK8* transcripts; filled boxes: exons; dotted line: exon extension; horizontal line: intronic regions; *: novel splice site. (**g**) The altered or physiologic transcription products of the minigene vectors were differentiated by size. Agarose gel with canonical splice site usage (378 nucleotide transcript) in cDNA of control PBMCs transfected with wildtype (Mini wt) and usage of the novel splice site (453 nucleotide transcript) in cDNA of PBMCs transfected with the mutated minigene vector (Mini mut); GFP- and mock-transfected as negative controls.

With this inconclusive WGS analyses and the clinical presentation of patient II.2 partly suggesting DOCK8-HIES, we reassessed *DOCK8* by Sanger sequencing. Sequencing *DOCK8* cDNA of patient II.2′s T cell blasts revealed double peaks downstream of exon 36 (Fig. 3a). We identified these double peaks as result of a combination of physiologic *DOCK8* transcripts and transcripts with exon 36 extended by 75 nucleotides at the 3′ end resulting in a premature stop codon after 18 nucleotides (Fig. 3a,b). We consulted the GTEx dataset^21,22^ for alternatively spliced *DOCK8* transcripts with extension of exon 36 and found rare events of exon extension by 16 or 133 nucleotides as visualized by Sashimi plot and the corresponding percent spliced in (psi) analysis (Supplementary Fig. S3). Although these rare events of extended exons suggest susceptibility of the intron downstream of exon 36 to splicing, an alternatively spliced *DOCK8* transcript with extension of exon 36 by 75 nucleotides was not present in the dataset. gDNA sequencing of the introns flanking exon 36 of patient II.2 and re-analyzing the WGS data identified a homozygous intronic nucleotide exchange (c.4626 + 76 A > G) introducing a novel splice site in *DOCK8* (Fig. 3a).

Both parents and the healthy sister were heterozygous for the c.4626 + 76 A > G variant which has not been reported in the databases dbSNP^23^ and gnomAD^24^. Droplet digital PCR (ddPCR) confirmed the cDNA sequencing results of patient II.2 (Fig. 3c) showing almost equal parts of wildtype *DOCK8* transcripts and altered elongated transcripts. To evaluate if expression of about 50% wildtype *DOCK8* transcript is sufficient to prevent a DOCK8-HIES phenotype, we performed ddPCR analyses with *DOCK8* cDNA of T cell blasts of healthy carriers of a heterozygous *DOCK8* c.3120 + 1 G > T mutation resulting in skipping of exon 25. All three healthy carriers had equal amounts of wildtype and truncated *DOCK8* transcripts suggesting that the *DOCK8* gene is haplosufficient (Fig. 3d).

In patient II.3 the homozygous intronic *DOCK8* alteration (c.4626 + 76 A > G) as in patient II.2 was identified soon after birth (Fig. 3a). In contrast to patient II.2, in patient II.3 all *DOCK8* transcripts showed the alteration with extension of exon 36 (Fig. 3a,c). Additionally, exon 32 was missing in about half of patient II.3′s *DOCK8* transcripts. gDNA sequencing of both flanking introns of exon 32 revealed no genetic alteration in patient II.3. Due to haplosufficiency of *DOCK8* we concluded that exon 32 skipping was not the cause of disease in patient II.3. This was supported by the fact that exon 32 skipping was also found as a rare event in the GTEx dataset^21,22^ as visualized by Sashimi plot and the corresponding psi analysis (Fig. 3e, Supplementary Fig. S3).

Since the intronic variant c.4626 + 76 A > G results in a novel donor splice site (AT > GT) in the intron between exon 36 and 37 of *DOCK8* and as *DOCK8* transcripts were differently affected in the two patients we assessed splice site usage by *in silico* analyses. Splice site prediction tools calculated higher scores for the novel donor splice site compared to the canonical splice site (NNSPLICE0.9^25^ score: 0.97 and 0.86; HSF^26^ score: 86.53 and 80.99; SplicePort^27^ score: 0.58 and 0.14 of novel splice site and canonical splice site, respectively). Moreover, SpliceAid2^28^ predicted the loss of multiple binding sites for splicing factors as a result of the c.4626 + 76 A > G alteration indicating a reduction of splicing activity at the canonical splice site. Thus, these bioinformatics analyses indicated the variant as a stronger donor site that outperforms the canonical donor site leading to altered transcripts. To verify the *in silico* prediction *in vitro* we introduced a minigene vector containing the intronic variant c.4626 + 76 A > G or the wildtype sequence into PBMCs of healthy individuals (Fig. 3f). In presence of the novel splice site, we solely detected altered transcripts with exon extension in PBMCs of healthy controls (Fig. 3g). Thus, the minigene analysis supported the exclusive expression of altered transcripts with an extended exon 36 as observed in patient II.3.

### DOCK8 expression analyses

**Figure 4:**
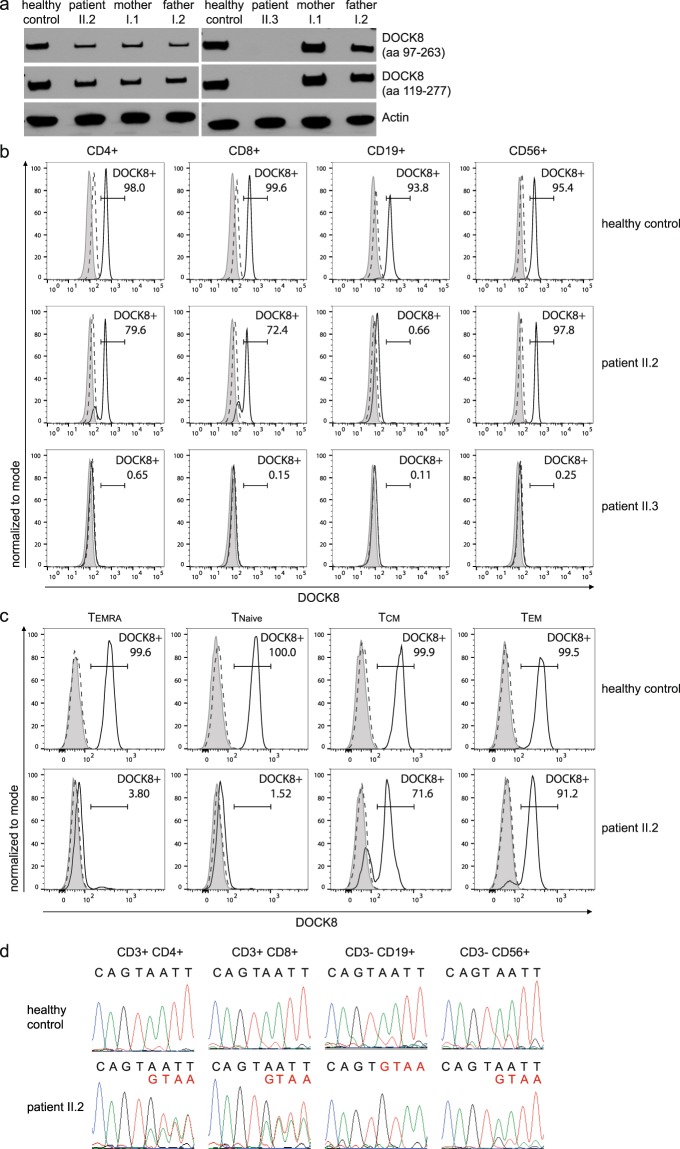
DOCK8 expression analysis. (**a**) Western blot analysis of whole PBMC lysates shows DOCK8 expression in patient II.2 and not in patient II.3 with two different DOCK8 antibodies (immunogen indicated in brackets; aa: amino acid); Actin as a loading control. Full-length western blots are provided in the Supplementary Appendix (Supplementary Fig. S4). (**b**) Flow cytometry of patient II.2 showed DOCK8 expression in majority of NK cells and T cells but no DOCK8 expression in B cells. All cell subsets of patient II.3 lack DOCK8 expression. Gray area: unstained; dashed line: isotype control; solid line: DOCK8 staining. (**c**) T cell subsets defined by naïve T cells (CCR7^+^CD45RA^+^), central memory T cells (CCR7^+^CD45RA^−^), effector memory T cells (CCR7^−^CD45RA^−^) and T_EMRA_ cells (CCR7^−^CD45RA^+^) showed no DOCK8 expression in T_EMRA_ and naïve T cells and DOCK8 expression in majority of central and effector memory T cells of patient II.2 compared to DOCK8 expression in all T cell subsets of a healthy control. (**d**) Sequencing of cDNA reveals double peaks in chromatograms of T and NK cells of patient II.2 indicating wildtype (black letters) and altered (red letters) transcripts. cDNA chromatogram of B cells shows only single peaks indicating altered transcripts.

The c.4626 + 76 A > G alteration found in patients II.2 and II.3 leads to a premature stop codon in the DOCK8 protein and consequently a lack of DOCK8 protein as observed in western blot and flow cytometric analyses in PBMCs of patient II.3 (Fig. 4). Western blot analysis of PBMCs of patient II.2, however, showed expression of DOCK8 protein (Fig. 4a, Supplementary Fig. S4). Detailed lymphocyte subset analysis revealed no DOCK8 protein expression in B cells of patient II.2, whereas most NK cells expressed DOCK8 (Fig. 4b). The majority of CD4^+^ and CD8^+^ T cells expressed DOCK8, while about 20–30% were DOCK8 negative. Naïve T cells and the majority of T_EMRA_ cells did not express DOCK8, whereas most effector memory T cells did express DOCK8 protein (Fig. 4c). The presence of DOCK8 protein in cell subsets corresponded with the transcript analysis of sorted lymphocyte subsets of patient II.2: B cells had only altered transcripts with exon extension in accordance with a missing expression of DOCK8 protein (Fig. 4d); NK and T cells expressed wildtype and altered *DOCK8* transcripts.

**Figure 5:**
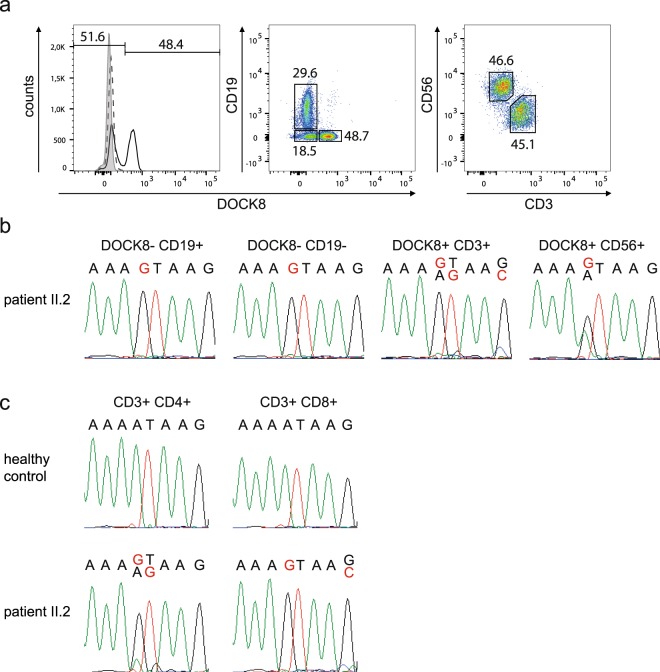
Analysis of somatic alterations in *DOCK8*. (**a**) Gating strategy to sort lymphocyte subsets according to their DOCK8 expression. PBMCs of patient II.2 were gated for lymphocytes and then DOCK8-negative cells into B cells (DOCK8^−^CD19^+^) and non-B cells (DOCK8^−^CD19^−^), and DOCK8-positive cells into T cells (DOCK8^+^CD19^−^CD3^+^) and NK cells (DOCK8^+^CD19^−^CD56^+^). (**b**) gDNA sequence of sorted cells of patient II.2 had a homozygous peak for the c.4626 + 76 A > G alteration (red letter) in T and B cells and a double peak with altered (red letter) and wildtype (black letter) sequence in NK cells. (**c**) gDNA sequence of unfixed and unpermeabilized PBMCs of patient II.2 sorted according to the lymphocyte subsets CD4^+^ and CD8^+^ T cells showing double peaks with altered (red letter) and wildtype (black letter) sequence in CD4^+^ T cells at positions c.4626 + 76 and c.4626 + 77 and at position c.4626 + 80 in CD8^+^ T cells.

To explain the expression of wildtype *DOCK8* transcript and DOCK8 protein in NK and T cells of patient II.2, we performed gDNA sequencing of sorted cells. Cells of patient II.2 were subdivided first by their DOCK8 expression and subsequently by different lymphocyte subsets (Fig. 5a). B cells (DOCK8^−^CD19^+^) and DOCK8-negative non-B cells (DOCK8^−^CD19^−^) were homozygous for the novel splice site (Fig. 5b). DOCK8-expressing cells were divided into T cells (DOCK8^+^CD19^−^CD3^+^) and NK cells (DOCK8^+^CD19^−^CD56^+^). A double peak for the mutated and the wildtype sequence was detected in DOCK8-expressing NK cells; hence, likely explaining DOCK8 protein expression in the majority of NK cells by somatic gDNA reversion to wildtype sequence. DOCK8-positive T cells showed in addition to the novel splice site small double peaks at position c.4626 + 76, c.4626 + 77, and c.4626 + 80.

Further sorting of T cell subsets explained DOCK8 protein re-expression in CD4^+^ T cells by somatic alterations at position c.4626 + 76 and c.4626 + 77 reverting the sequence to wildtype or destroying the novel splice site (Fig. 5c). In the presence of the somatic variant c.4626 + 80 G > C splice site prediction tools calculated lower scores for the novel splice site (NNSPLICE0.9^25^ score: 0.97 and 0.10; HSF^26^ score: 86.53 and 74.52; SplicePort^27^ score: 0.58 and −0.56 of novel splice site without and in the presence of the variant c.4626 + 80 G > C). Hence, DOCK8 expression in CD8^+^ T cells resulted from reuse of the physiologic splice site due to weakening of the novel splice site by the somatic variant c.4626 + 80 G > C. Moreover, SpliceAid2^28^ predicted a novel binding site of the splicing activator DAZAP1^29^ as a result of the c.4626 + 80 G > C alteration indicating an increase of splicing activity at the canonical splice site.

## Discussion

Our experience of two siblings with HIES findings shows the complex process of a molecular genetic diagnosis including verification of the identified homozygous intronic *DOCK8* variant c.4626 + 76 A > G as disease-causing in the presence of somatic alterations. Hyperextensible joints and impaired Y705-STAT3 phosphorylation pointed towards STAT3-HIES in patient II.2^1,2,5,6^. But in contrast to STAT3-HIES patients, who present with a reduction of Th17 cell counts due to diminished STAT3 function^8,9^, the patient II.2 presented with normal Th17 cell counts. To explain her normal Th17 cell counts despite a STAT3 phosphorylation defect, we revealed intact IL21-induced STAT3 activation in patient II.2′s lymphocytes as an alternative route for Th17 differentiation^30,31^. Her recurrent viral infections, high eosinophil counts, multiple food allergies, and failure to thrive raised suspicion of DOCK8-HIES^3–5,11^, while the lack of lymphopenia, rather normal to high lymphocyte counts, and particularly the DOCK8 protein expression in PBMCs argued against DOCK8-HIES. However, assessment of *DOCK8* transcripts and subsequent gDNA sequencing of intronic regions by Sanger technique identified the homozygous intronic *DOCK8* variant c.4626 + 76 A > G, creating a novel splice site, retrospectively also found in the NGS data. During the time of identifying this variant, the second affected child (patient II.3) was born carrying the same homozygous *DOCK8* alteration yet missing DOCK8 protein expression in her PBMCs. *In silico* and *in vitro* analyses showed that the novel splice site was preferentially used by the spliceosome regardless of the presence of the canonical splice site, confirming the variant c.4626 + 76 A > G as disease-causing. Hence, the exclusive use of the novel splice site explained the lack of DOCK8 protein in PBMCs of patient II.3 and B cells of patient II.2.

Yet why was DOCK8 protein expressed in NK cells and parts of T cells of patient II.2? Since somatic reversions have been reported in DOCK8-HIES^32–34^, we investigated *DOCK8* transcripts in NK cells and T cell subsets confirming our hypothesis of somatic alterations. Consequently, most of patient II.2′s T cells were able to produce wildtype *DOCK8* transcripts likely leading to a survival advantage and clonal expansion of DOCK8 expressing T cells under selective pressure, e.g. exerted by viral infections, as reported for DOCK8-HIES and other PIDs^32,34,35^.

This selective advantage of DOCK8 expressing T cells may also explain why we did not observe a more pronounced shift from memory CD8^+^ T cells to T_EMRA_ cells in patient II.2, as reported to be characteristic of DOCK8-HIES patients due to reduced memory cell persistence^36^. We suggest that the DOCK8 expression, observed in the majority of her CD8^+^ effector memory T cells, rescued memory cell persistence and resulted in a reduced shift towards a T_EMRA_ phenotype. The dependence of memory T cell persistence on DOCK8 expression was further supported by the fact that the majority of her T_EMRA_ cells were DOCK8 negative. The skewed T cell distribution of more than 80% effector memory and T_EMRA_ cells of total T cells likely also explains the impaired IL6-induced STAT3 activation in lymphocytes of patient II.2. Only naïve T cells and a few central memory T cells are reported to show STAT3 phosphorylation after IL6 stimulation, whereas in effector memory T cells and T_EMRA_ cells STAT3 is not activated by IL6 stimulation^37–39^. Furthermore, IL6 does not phosphorylate STAT3 in B and NK cells, whereas IL10 induces STAT3 phosphorylation in all lymphocyte subsets^40^. Hence, once T cell subpopulations, including naïve T cells, normalized in patient II.2 after HSCT, IL6-induced STAT3 phosphorylation also normalized and was therefore not caused by the *DOCK8* alteration but secondarily caused due to shifted T cell subsets.

Taken together, HSCT cured patient II.3, who was diagnosed early, while patient II.2 still requires intensive treatment underscoring the need for and the benefit of an early diagnosis. The fact that the autosomal recessive WGS analysis did not prioritize the disease-causing alteration and that within the GTEx dataset^21,22^ we found evidence that *DOCK8* exon 36 is susceptible to alternative splicing demonstrates, that integrating existing RNA sequencing data into WES and WGS analyses may help to prioritize variants in introns susceptible to alternative splicing^41^. To date, transcriptomics is often used as a complementary diagnostic tool if patients remain without molecular diagnosis after WES or WGS^41,42^. However, molecular diagnosis might have been delayed even with additional transcriptome analysis as patient II.2 expressed altered and due to somatic alterations wildtype *DOCK8* transcripts.

In conclusion, the complex diagnostic process of these patients shows how somatic alterations compromise molecular diagnostics and reminds to keep the clinical presentation in mind calling for a close interaction between clinicians, scientists, and bioinformaticians.

## Material and Methods

Patients, their healthy parents, molecularly defined DOCK8-HIES patients, healthy DOCK8-HIES carriers, and healthy controls were included in this study after written informed consent given by patients or their legal guardians. Patients’ clinical, immunologic, and molecular findings were assessed by Next Generation Sequencing (NGS), expression and splicing analysis, and cell phenotyping and compared to controls as indicated; for detailed methods see Supplementary Materials and Methods. The study was approved by the local reviewing board (Ethikkommission bei der Medizinischen Fakultät der Ludwig-Maximilians-Universität München, #381-13), written informed consent was obtained. All research was performed in accordance with relevant guidelines and regulations.

## Electronic supplementary material

Supplementary Appendix

## Data Availability

The datasets generated and analyzed during this study are not publicly available due to country specific restrictions on data safety protecting the privacy of the involved study participants.
