# Supplementary material for: Somatic alterations compromised molecular diagnosis of DOCK8 hyper-IgE syndrome caused by a novel intronic splice site mutation
Source: Sci Rep. 2018 Nov 13;8:16719. doi: 10.1038/s41598-018-34953-z (PMC6233225; doi:10.1038/s41598-018-34953-z)
Supplement: Supplementary file 1 — Supplementary Appendix [file 41598_2018_34953_MOESM1_ESM.pdf]

Somatic alterations compromised molecular diagnosis of DOCK8 hyper-IgE syndrome caused by a novel intronic splice site mutation

Beate Hagl PhD[1,2], Benedikt D Spielberger MD[1,2], Silvia Thoene PhD[3,4,5], Sophie Bonnal PhD[6,7], Christian Mertes MSc[8], Christof Winter MD, PhD[3,4,5], Isaac J Nijman PhD[9], Shira Verduin MSc[9], Andreas C Eberherr MSc[1], Anne Puel PhD[10,11,12], Detlev Schindler MD[13], Jürgen Ruland MD[3,4,5,14], Thomas Meitinger MD, PhD[15], Julien Gagneur PhD[8,16], Jordan S Orange MD, PhD[17,18,19], Marielle E van Gijn PhD[9], Ellen D Renner MD[1,2,20,*]

[1]Chair and Institute of Environmental Medicine, UNIKA-T, Technical University of Munich and Helmholtz Zentrum Munich, Munich/Augsburg, Germany; [2]University Children's Hospital, Dr. von Haunersches Kinderspital, Ludwig Maximilian University, Munich, Germany; [3]Institute of Clinical Chemistry and Pathobiochemistry, Klinikum rechts der Isar, Technical University of Munich, Munich, Germany; [4]German Cancer Consortium (DKTK), partner site Munich, Munich, Germany; [5]German Cancer Research Center (DKFZ), Heidelberg, Germany; [6]Centre for Genomic Regulation (CRG), The Barcelona Institute of Science and Technology, Dr. Aiguader 88, Barcelona 08002, Spain; [7]Universitat Pompeu Fabra (UPF), Barcelona, Spain; [8]Department of Informatics, Technical University of Munich, Garching, Germany; [9]Department of Genetics, University Medical Center Utrecht, Utrecht, The Netherlands; [10]Laboratory of Human Genetics of Infectious Diseases, Necker Branch, Necker Medical School, Paris, France; [11]Paris Descartes University, Sorbonne Paris Cité, Institut Imagine, Paris, France; [12]St Giles Laboratory of Human Genetics of Infectious Diseases, Rockefeller Branch, Rockefeller University, New York, NY, USA; [13]Department of Human Genetics, University of Würzburg, Würzburg, Germany; [14]German Center for Infection Research (DZIF), partner site Munich, Munich, Germany; [15]Institute of Human Genetics, Technical University of Munich and Helmholtz Zentrum Munich, Neuherberg, Germany; [16]Quantitative Biosciences Munich, Gene Center, Department of Biochemistry, Ludwig Maximilian University, Munich, Germany; [17]Center for Human Immunobiology of Texas Children's Hospital/Department of Pediatrics, Baylor College of Medicine, Houston, TX, USA; [18]Department of Pediatrics, Division of Immunology, Allergy, and Rheumatology, Baylor College of Medicine, and Texas Children's Hospital, Houston, TX, USA; [19]Department of Pediatrics, Baylor College of Medicine, and Texas Children's Hospital, Houston, TX, USA; [20]Hochgebirgsklinik and Christine-Kühne-Center for Allergy Research and Education (CK-Care), Davos, Switzerland
B.H. and B.D.S. contributed equally to this work

SUPPLEMENTARY APPENDIX

TABLE OF CONTENTS:

Supplementary Material and Methods **3**

Patients, clinical and immunologic work-up 3

Genetic analyses 3

DOCK8 mRNA expression analyses 4

DOCK8 protein assessment 4

DOCK8 splicing analyses 5

Supplementary Figures **6**

Supplementary Figure S1: Autoantibody analysis 6

Supplementary Figure S2: ARHGAP32 analysis 7

Supplementary Figure S3: *In silico* analysis of preexisting RNA sequencing data 8

Supplementary Figure S4: DOCK8 western blot analysis 10

References **12**

Supplementary Material and Methods:

Patients, clinical and immunologic work-up

A consanguineous family of Turkish descent with two children with HIES findings and a healthy sibling was assessed. The study was approved by the local reviewing board (Ethikkommission bei der Medizinischen Fakultät der Ludwig-Maximilians-Universität München, #381-13), written informed consent of patients or their legal guardians was obtained. All research was performed in accordance with relevant guidelines and regulations.

Differential blood count and serum immunoglobulin level were assessed as to standardized protocols. We isolated peripheral blood mononuclear cells (PBMCs) from venous blood using Biocoll Separating Solution (BIOCHROM AG, Berlin, Germany). Lymphocyte stimulation with an antigen mixture and different mitogens was performed as previously described[1].

Lymphocyte subsets were defined by total T cells ($CD3^+$), T helper cells ($CD3^+CD4^+$), cytotoxic T cells ($CD3^+CD8^+$), Th17 cells ($CD3^+CD4^+IL17^+IFNgamma^-$), total B cells ($CD19^+$), memory B cells ($CD19^+CD27^+$), and NK cells ($CD16^+CD56^+$), stained with the according antibodies, and analyzed by flow cytometry (BD FACSCalibur and BD LSRFortessa, BD Biosciences, San Jose, CA, USA) as previously described.[1] Subdivided T cell subsets were defined by naïve T cells ($CD3^+CCR7^+CD45RA^+$), central memory T cells ($CD3^+CCR7^+CD45RA^-$), effector memory T cells ($CD3^+CCR7^-CD45RA^-$) and T_{EMRA} cells ($CD3^+CCR7^-CD45RA^+$) and stained according to the following protocol adapted from [2]. Briefly, cells were treated with Biolegend Aqua Zombie dye BV510 to exclude dead cells according to the manufacturer's instruction. After staining with anti-CCR7 BV421, anti-CD3 APC-H7, anti-CD4 BV711, anti-CD8 PE, anti-CD19 PE-Cy7, anti-CD56 APC, anti-CD45RA BV650 (all BD) cells were analyzed by flow cytometry. Patients' values were compared to age-matched references as previously described[1] or to healthy individuals.

STAT3 tyrosine phosphorylation was assessed in PBMCs by flow cytometry using the BD Phosflow reagents per the manufacturer's instruction (BD Biosciences) and by western blot using antibodies to Phospho-STAT3 (Tyr705), STAT3 and beta-Actin (all Cell Signaling, Danvers, MA, USA) as previously described.[3] To analyze a putative effect of autoantibodies on STAT3 phosphorylation, PBMCs of a healthy control were incubated overnight in the absence or presence of 10% patient or different control sera (adopted from [4]) and Phospho-STAT3 (Tyr 705) was measured by flow cytometry after IL6 and IL10 stimulation. The effect of ARHGAP32 on STAT3 phosphorylation was assessed by transfecting a wildtype *ARHGAP32* vector (OriGene, Rockville, MD, USA) into healthy control PBMCs by nucleofection using the Human T Cell Nucleofector Kit and a Nucleofector 2b (both Lonza, Basel, Switzerland) followed by flow cytometric analysis as well as by assessing STAT3 phosphorylation in HAP1 ARHGAP32 knock-out cells and wildtype HAP1 cells (Horizon, Cambridge, UK) by western blot.

Genetic analyses

Targeted next-generation sequencing was performed as previously described[5]. Sample prep (TruSeq DNA, input 250 ng) and the Illumina's HiSeqX sequencing platform were used according to manufacturer's instructions to perform WGS. Illumina data were processed with the inhouse developed pipeline v1.2.1 (https://github.com/UMCUGenetics/IAP) including GATK v3.2.2[6] according to the best practice guidelines.[7,8] Briefly, paired end reads were mapped with BWA-MEM v0.7.5a[9] to GRCh37, duplicates marked, lanes merged, and indels realigned. Base Quality Score recalibration was left out since it did not improve our results significantly. Next, GATK Haplotypecaller was used to call SNPs and indels to create GVCFS. These GVCFS were genotyped with GATK's GenotypeGVCFs for the described family. Variants were flagged as PASS if none of the following criteria was fulfilled: QD<2.0,

MQ<40.0, FS>60.0, HaplotypeScore>13.0, MQRankSum<-12.5, ReadPosRankSum<-8.0, snpclusters>=3 in 35bp. For indels: QD<2.0, FS>200.0, ReadPosRankSum<-20.0. Effect predictions and annotation was added using snpEFF[10] and dbNSFP[11]. De-novo variants were detected with GATKs' phase-by-transmission and filtering the Mendelian violations on the de-novo model and coverage >10x for every call.
Sanger sequencing of the coding region and intron-exon boundaries of the *DOCK8* gene was performed on gDNA and cDNA level using specific oligonucleotide primers, as previously described[3]. Primer sequences are available upon request. Amplified gene fragments were sequenced with an ABI 3730 capillary sequencer (Applied Biosystems, Carlsbad, CA, USA). Mutations were reported using the nomenclature of den Dunnen and Antonarakis.[12]

DOCK8 mRNA expression analyses

Expression levels of DOCK8 splice variants were quantified by an intercalating dye (EvaGreen)-based approach in 96-well plates on a QX200 droplet digital PCR (ddPCR) system with automatic droplet generation (Bio-Rad Laboratories) in duplicates with reaction volumes of 21 µl, cDNA input of 5-20 ng RNA equivalent, and the following cycling conditions: 5 min at 95 °C, 40 cycles of (30 s at 96 °C, 1 min at 60 °C), 5 min at 4 °C, and 5°min at 90 °C. The following primers (synthesized by Integrated DNA Technologies, IDT) were used at a final concentration of 100 nM: DOCK8.36for: 5-TGC CAC CCT TTA CCT CCT CA-3, DOCK8.37rev: 5-TTC CCA CCA AAG ATG CCA G-3, DOCK8.24for: 5-GCC TGG TTC TTC TTT GAG CTT C-3, DOCK8.26rev: 5-AGA AAG CCA GGC TGA TGT TCA T-3. All ddPCR runs were performed with cDNA of patients or healthy carriers expressing the splice variant (verified by Sanger sequencing), cDNA of healthy individuals as negative control, and purified, nuclease-free water as no-template control (NTC). Droplet fluorescence intensity values were exported from QuantaSoft (Bio-Rad Laboratories). Custom scripts were developed and used to import the intensity values into R (version 3.2.3; http://www.r-project.org). To compensate for baseline shifts of fluorescence intensity between reaction wells, data were centered using the following procedure. First, the droplet with the highest fluorescence intensity value in NTC wells was identified and its fluorescence intensity was denoted as maxNTC. Next, droplets in each well were divided into two groups with either low (≤ 2*maxNTC) or high (> 2*maxNTC) intensity. For each well, the median intensity value of the low intensity group of droplets (medianLow) was calculated. Then, for each well, droplet intensity values were normalized by subtracting medianLow from each intensity value. Droplet intensity values were plotted after each step and inspected for negative and positive clusters. In order to automatically assign droplets to one of three groups (negative, positive for splice variant 1, or positive for splice variant 2), k-means clustering with pre-specified number of clusters k was performed for each well.
Target mRNA concentrations c were then calculated for each well from the number of positive droplets Np and negative droplets Nn and the average droplet volume V = 0.85 nanoliter based on Poisson distribution statistics using the formula $c = (ln(Np + Nn) - ln(Nn))/V$, where ln is the natural logarithm. For each splice variant, only droplets positive for this particular splice variant were considered positive, and all other droplets were considered negative.

DOCK8 protein assessment

To assess DOCK8 protein expression in different lymphocyte subsets flow cytometry was performed adapted from[13] using the following antibodies: anti-CD3 APC-H7, anti-CD4 BV711, anti-CD8 PE, anti-CD19 PE-Cy7, anti-CD56 APC (all BD), anti-DOCK8 (SantaCruz), isotype control (Biolegend), and FITC rat anti-mouse IgG (Biolegend). DOCK8 western blot analyses were performed on cells lysed with complete Lysis-M EDTA-free solution (Roche Applied Science, Penzberg, Germany). Following SDS-

PAGE with NuPAGE Novex 3%-8% Tris-Acetate Gels and transfer with the NuPAGE Large Protein Blotting Kit (Invitrogen, Carlsbad, CA, USA) blots were probed with antibodies to DOCK8 (Abcam, Cambrige, UK and Santa Cruz, Starr County, Texas, USA) and beta-Actin (Cell Signaling, Danvers, MA, USA) and developed with secondary horseradish peroxidase conjugated polyclonal antibodies (Invitrogen, Carlsbad, CA, USA) and SuperSignal West Femto Maximum Sensitivity Substrate (Pierce, Rockford, IL, USA). Spectra Multicolor High Range Protein Ladder (ThermoFisher Scientific, Waltham, MA, USA) was used as size standard.

DOCK8 splicing analyses

Prior to the Sashimi plot and the percent spliced in (psi, Ψ) analysis, the GTEx samples were filtered to obtain a more homogeneous dataset.[14,15] 2616 samples passed the following filter steps: assembly=="HG19_Broad_variant", library_type=="cDNAShotgunStrandAgnostic", samples are no technical controls, and molecular_data_type=="Allele-Specific Expression". All reads over all samples were pooled together and only reads that mapped around the DOCK8 exons 32 and 36 were included in the Sashimi plots. To evaluate how efficient an intron is spliced out of a transcript, a psi analysis was performed. The Ψ values for the 5' and 3' sites were calculated for each sample as: $\Psi_5(D,A) = \frac{n(D,A)}{\sum_{A'} n(D,A')}$ and $\Psi_3(A,D) = \frac{n(D,A)}{\sum_{D'} n(D',A)}$, where *D* is a donor site and *A* is an acceptor site.[16] *n(D,A)* denotes the number of reads spanning the given junction D-A. The statistic is based on split reads, which are reads spanning multiple exons and having at least one gap in the alignment, the spliced intron. Here the 5' percent spliced in (Ψ_5) value is calculated as the fraction of split reads covering the intron of interest over all split reads covering the given donor site. The 3' percent splice in (Ψ_3) value is calculated the same way but taking the reads covering the acceptor site.

In silico analysis of splice site prediction was performed by utilizing multiple methods: NNSPLICE0.9, Human Splicing Finder (HSF) Version 3.1, SpliceAid2, SplicePort, and CryptSplice.[17-21] CryptSplice did not obtain results since the variant was too far away from the canonical splice site. Splicing was evaluated by minigene assay. In brief: PBMCs of healthy controls were transiently transfected with a wildtype and a mutated minigene plasmid using the human T-cell nucleofector kit (Lonza, Cologne, Germany) according to manufacturer's instructions. Minigene constructs were generated by cloning a PCR amplicon of human genomic DNA into a pCMV56 vector as previously described.[22,23] The introduced PCR amplicon started at the first nucleotide of exon 36 to position 25 downstream of exon 37 of *DOCK8* and was flanked by sequence tags to differentiate minigene transcripts from endogenous DOCK8 transcripts. Transcripts were reverse transcribed using the ProtoScript II First Strand cDNA Synthesis Kit and cDNA was amplified by PCR using the LongAmpTaq 2x Master Mix (both New England Biolabs, Ipswich, MA, USA) according to manufacturer's instruction. The following cycling conditions were used: 3 min at 94 °C, 25 cycles of (30 s at 94 °C, 30 s at 60 °C, 25 s at 65 °C), 10 min at 65 °C, and hold at 4 °C.

Supplementary Figures:

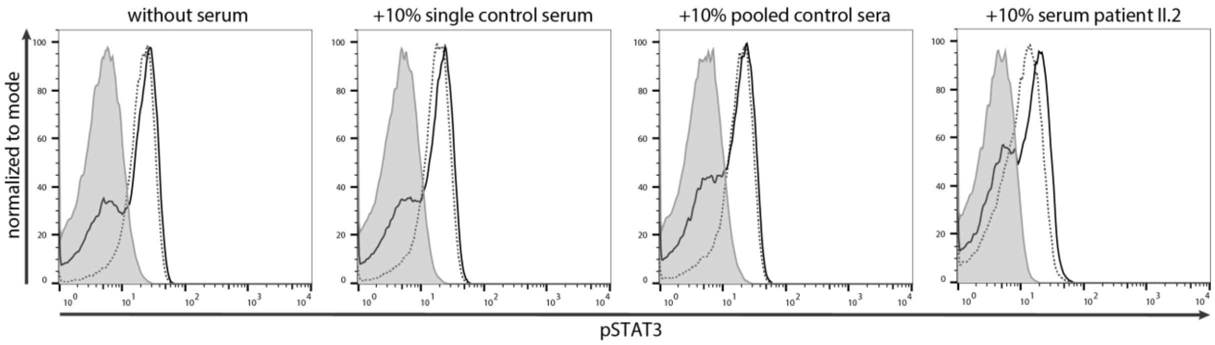

Supplementary Figure S1: Autoantibody analysis.

Flow cytometric analysis showing comparable Y705-STAT3 phosphorylation of lymphocytes after 20 min. stimulation with 200 ng/ml IL6 (solid line) or IL10 (dotted line), incubated with 10% patient or control sera overnight; pooled control sera: sera of seven different healthy controls; filled gray area: unstimulated lymphocytes.

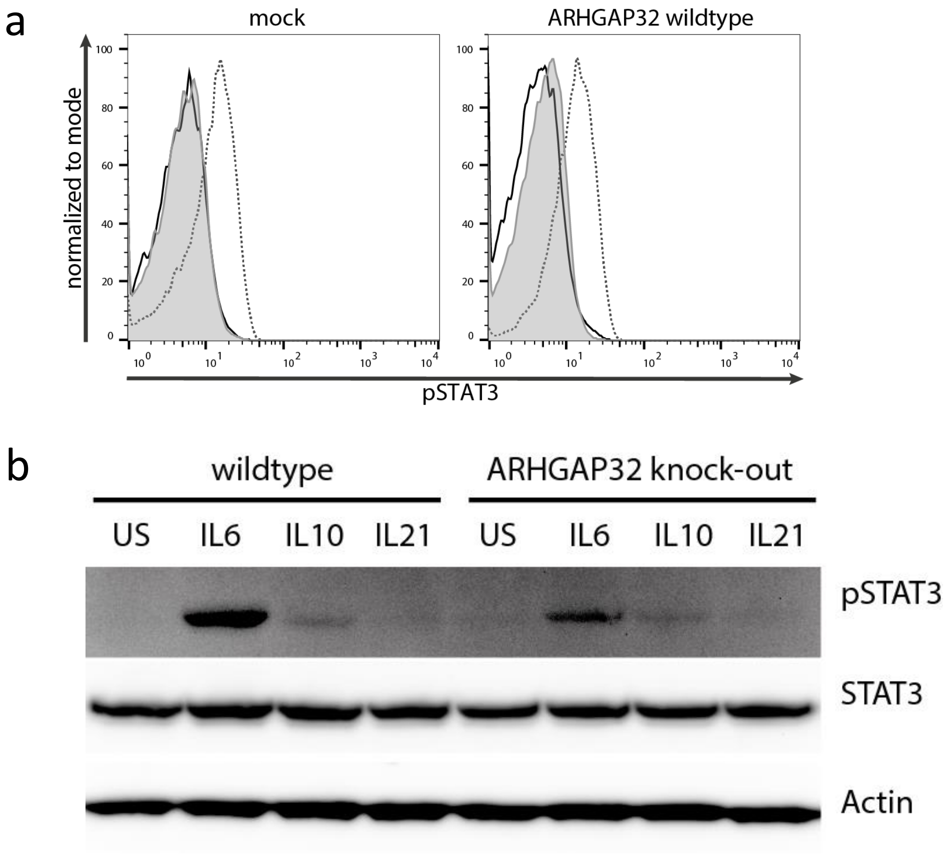

Supplementary Figure S2: ARHGAP32 analysis.

(a) Flow cytometric analysis of Y705-STAT3 phosphorylation of mock- and ARHGAP32-transfected PBMCs of patient II.2 with no rescue of IL6-induced (solid line) Y705-STAT3 phosphorylation by ARHGAP32 overexpression; filled gray area: unstimulated lymphocytes; dotted line: lymphocytes stimulated with IL10. PBMCs were stimulated 20 min. with 200 ng/ml IL6 or IL10. (b) Western blot analysis of whole cell lysates of wildtype and ARHGAP32 knock-out cells, unstimulated or 20 min. stimulated with 20 ng/ml IL6 or IL10, or 10 ng/ml IL21. Expression of STAT3 phosphorylated at Y705 (pSTAT3) and total STAT3 (STAT3) was assessed showing intact stimulation of STAT3 phosphorylation by IL6 in wildtype and ARHGAP32 knock-out cells and no distinct induction of STAT3 phosphorylation by IL10 or IL21 in both cell lines; Actin as loading control.

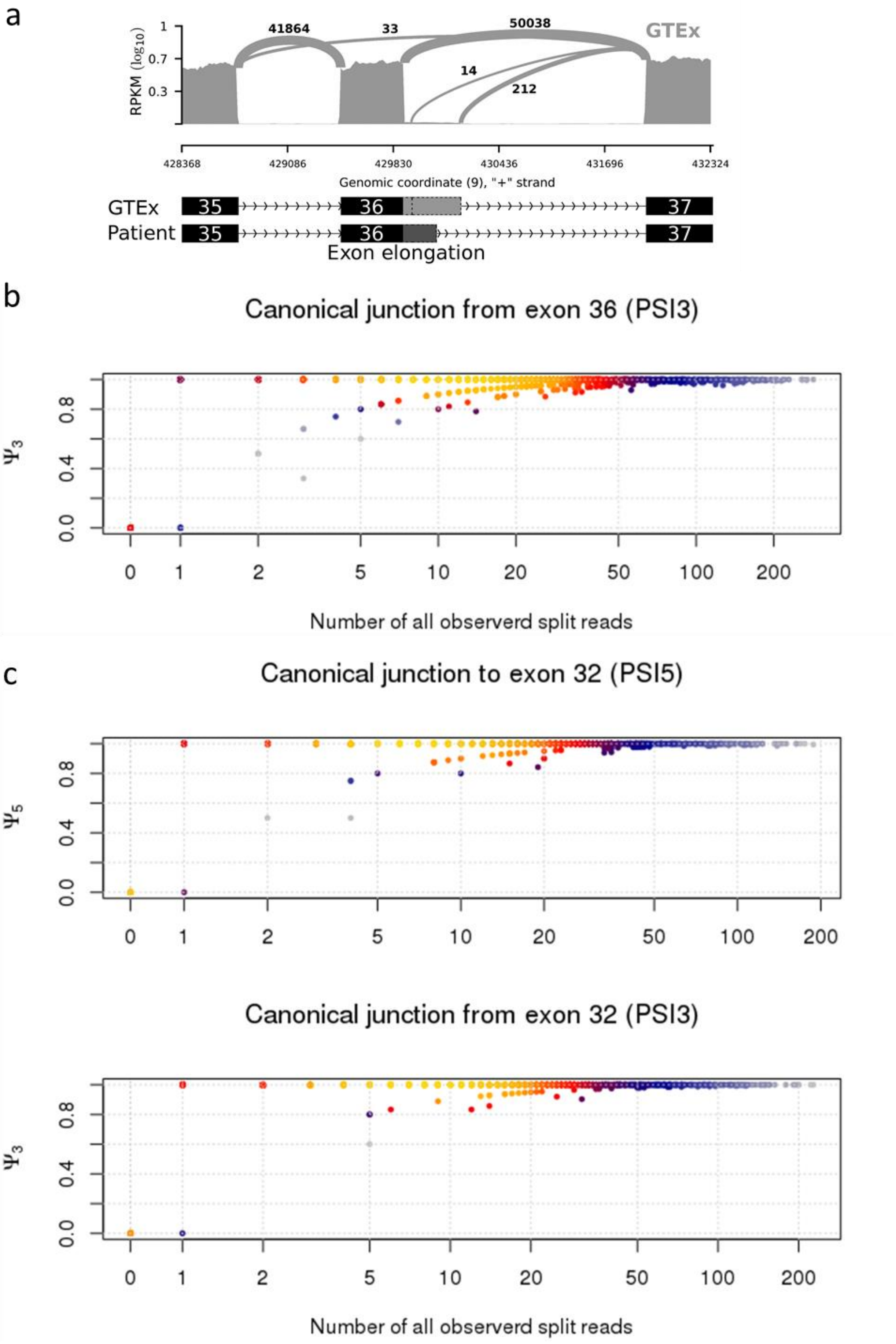
a
GTEx
RPKM (log10)
1
0.7
0.3
41864
33
50038
14
212
428368
429086
429830
430436
431696
432324
Genomic coordinate (9), "+" strand
GTEx
Patient
35
36
37
Exon elongation
b
Canonical junction from exon 36 (PSI3)
Ψ3
0.0
0.4
0.8
0
1
2
5
10
20
50
100
200
Number of all observerd split reads
c
Canonical junction to exon 32 (PSI5)
Ψ5
0.0
0.4
0.8
0
1
2
5
10
20
50
100
200
Canonical junction from exon 32 (PSI3)
Ψ3
0.0
0.4
0.8
0
1
2
5
10
20
50
100
200
Number of all observerd split reads

Supplementary Figure S3: ***In silico*** **analysis of preexisting RNA sequencing data.**

(a) Sashimi plot of RNA sequencing data based on GTEx samples[14,15] showing rare events of exon extension by 16 and 133 nucleotides; read counts accumulated over all samples. (b)(c) Heatscatter plots of Ψ_5 and Ψ_3 values and the total split read counts for all GTEx samples. On the x-axis the overall expression of *DOCK8* at the investigated site (number of all observed reads covering the splice site) is shown against the fraction of transcripts with a spliced intron of interest (Ψ_5/Ψ_3) on the y-axis. Each dot represents one sample. A Ψ value above 0.5 indicates that the intron of interest is spliced out in the majority of the transcripts, while a Ψ value below 0.5 indicates that other isoforms are more favored overall. (b) A heatscatter plot of Ψ_5 values for the donor site of the canonical exon junction between exon 36 and 37 is shown. (c) Heatscatter plots depicting the Ψ_5 and Ψ_3 values of the canonical exon junction between exon 31 and 32 and exon 32 and 33, respectively.

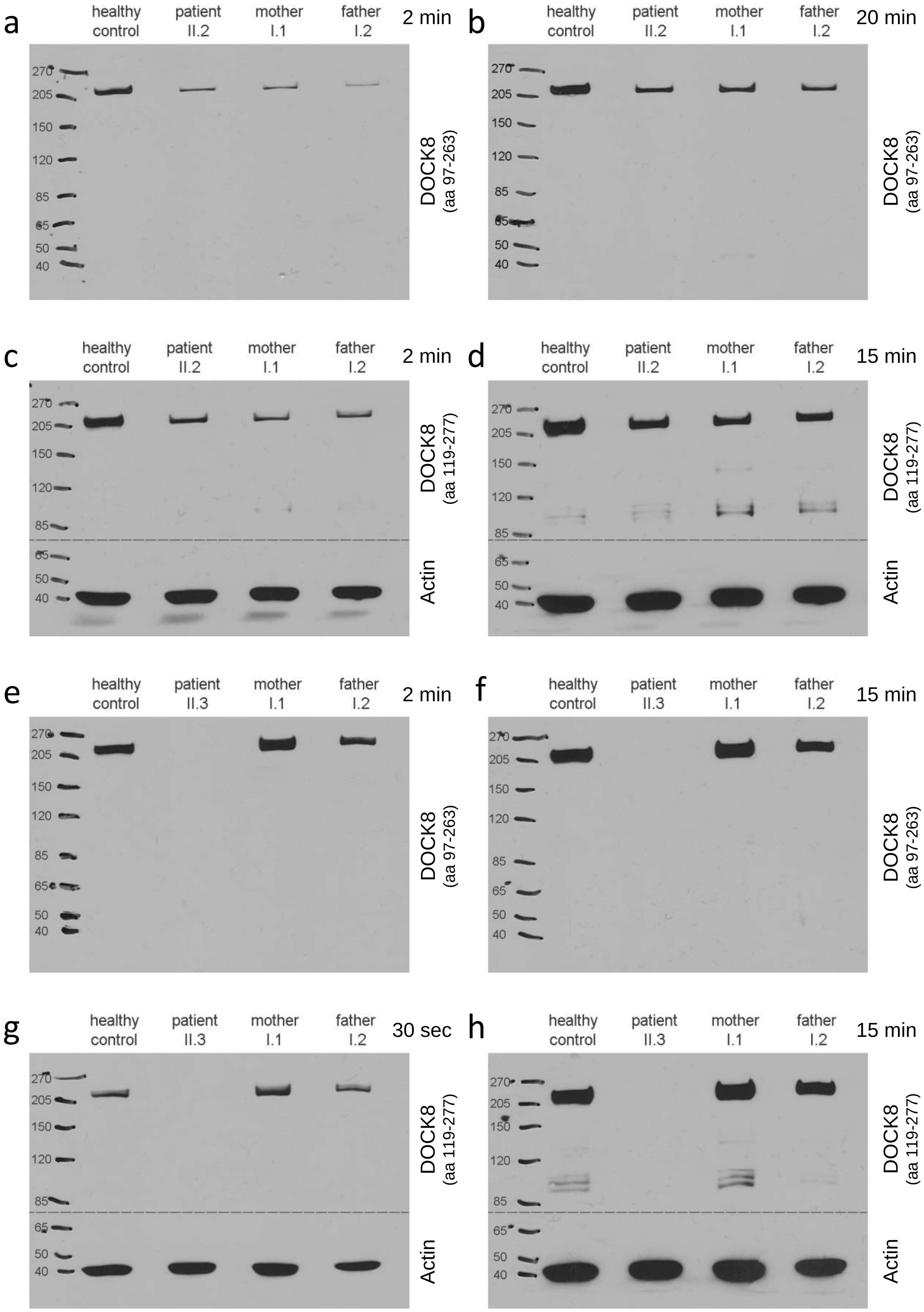

a
healthy control
patient II.2
mother I.1
father I.2
2 min
270
205
150
120
85
65
50
40
DOCK8 (aa 97-263)
b
healthy control
patient II.2
mother I.1
father I.2
20 min
DOCK8 (aa 97-263)
c
healthy control
patient II.2
mother I.1
father I.2
2 min
DOCK8 (aa 119-277)
Actin
d
healthy control
patient II.2
mother I.1
father I.2
15 min
DOCK8 (aa 119-277)
Actin
e
healthy control
patient II.3
mother I.1
father I.2
2 min
DOCK8 (aa 97-263)
f
healthy control
patient II.3
mother I.1
father I.2
15 min
DOCK8 (aa 97-263)
g
healthy control
patient II.3
mother I.1
father I.2
30 sec
DOCK8 (aa 119-277)
Actin
h
healthy control
patient II.3
mother I.1
father I.2
15 min
DOCK8 (aa 119-277)
Actin

Supplementary Figure S4: DOCK8 western blot analysis.

Full-length western blots of whole PBMC lysates of patient II.2 (a-d) and patient II.3 (e-h) in comparison to a healthy control and the parents are shown. Western blots were probed with different DOCK8 antibodies (immunogen indicated in brackets; aa: amino acid) and Actin as a loading control. Western blots were cut at the dashed lines where indicated (c, d, g, h). Exposure times are indicated; sec: seconds, min: minutes.

References:

1. Hagl B, Heinz V, Schlesinger A, et al. Key findings to expedite the diagnosis of hyper-IgE syndromes in infants and young children. Pediatr Allergy Immunol 2016;27:177-84.
2. Randall KL, Chan SS, Ma CS, et al. DOCK8 deficiency impairs CD8 T cell survival and function in humans and mice. J Exp Med 2011;208:2305-20.
3. Renner ED, Rylaarsdam S, Anover-Sombke S, et al. Novel signal transducer and activator of transcription 3 (STAT3) mutations, reduced T(H)17 cell numbers, and variably defective STAT3 phosphorylation in hyper-IgE syndrome. J Allergy Clin Immunol 2008;122:181-7.
4. Puel A, Picard C, Lorrot M, et al. Recurrent staphylococcal cellulitis and subcutaneous abscesses in a child with autoantibodies against IL-6. J Immunol 2008;180:647-54.
5. Nijman IJ, van Montfrans JM, Hoogstraat M, et al. Targeted next-generation sequencing: a novel diagnostic tool for primary immunodeficiencies. J Allergy Clin Immunol 2014;133:529-34.
6. McKenna A, Hanna M, Banks E, et al. The Genome Analysis Toolkit: a MapReduce framework for analyzing next-generation DNA sequencing data. Genome Res 2010;20:1297-303.
7. Van der Auwera GA, Carneiro MO, Hartl C, et al. From FastQ data to high confidence variant calls: the Genome Analysis Toolkit best practices pipeline. Curr Protoc Bioinformatics 2013;43:11 0 1-33.
8. van der Auwera GA. Best Practices for Variant Discovery in DNAseq. Broad Institute Genome Analysis Toolkit 2013;http://gatkforums.broadinstitute.org/gatk/discussion/3238/best-practices-for-variant-discovery-in-dnaseq.
9. Li H, Durbin R. Fast and accurate short read alignment with Burrows-Wheeler transform. Bioinformatics 2009;25:1754-60.
10. Cingolani P, Platts A, Wang le L, et al. A program for annotating and predicting the effects of single nucleotide polymorphisms, SnpEff: SNPs in the genome of Drosophila melanogaster strain w1118; iso-2; iso-3. Fly (Austin) 2012;6:80-92.
11. Liu X, Jian X, Boerwinkle E. dbNSFP: a lightweight database of human nonsynonymous SNPs and their functional predictions. Hum Mutat 2011;32:894-9.
12. den Dunnen JT, Antonarakis SE. Nomenclature for the description of human sequence variations. Hum Genet 2001;109:121-4.
13. Pai SY, de Boer H, Massaad MJ, et al. Flow cytometry diagnosis of dedicator of cytokinesis 8 (DOCK8) deficiency. J Allergy Clin Immunol 2014;134:221-3.
14. Consortium GT. The Genotype-Tissue Expression (GTEx) project. Nat Genet 2013;45:580-5.
15. Consortium GT. Human genomics. The Genotype-Tissue Expression (GTEx) pilot analysis: multitissue gene regulation in humans. Science 2015;348:648-60.
16. Pervouchine DD, Knowles DG, Guigo R. Intron-centric estimation of alternative splicing from RNA-seq data. Bioinformatics 2013;29:273-4.
17. Reese MG, Eeckman FH, Kulp D, Haussler D. Improved splice site detection in Genie. J Comput Biol 1997;4:311-23.
18. Desmet FO, Hamroun D, Lalande M, Collod-Beroud G, Claustres M, Beroud C. Human Splicing Finder: an online bioinformatics tool to predict splicing signals. Nucleic Acids Res 2009;37:e67.
19. Dogan RI, Getoor L, Wilbur WJ, Mount SM. SplicePort--an interactive splice-site analysis tool. Nucleic Acids Res 2007;35:W285-91.
20. Piva F, Giulietti M, Burini AB, Principato G. SpliceAid 2: a database of human splicing factors expression data and RNA target motifs. Hum Mutat 2012;33:81-5.
21. Lee M, Roos P, Sharma N, et al. Systematic Computational Identification of Variants That Activate Exonic and Intronic Cryptic Splice Sites. Am J Hum Genet 2017;100:751-65.
22. Forch P, Puig O, Kedersha N, et al. The apoptosis-promoting factor TIA-1 is a regulator of alternative pre-mRNA splicing. Mol Cell 2000;6:1089-98.
23. Sakamoto H, Inoue K, Higuchi I, Ono Y, Shimura Y. Control of Drosophila Sex-lethal pre-mRNA splicing by its own female-specific product. Nucleic Acids Res 1992;20:5533-40.
